# Cancer prevention and control: hepatocellular carcinoma

**DOI:** 10.3332/ecancer.2019.949

**Published:** 2019-07-25

**Authors:** Pierre Hainaut, Amina Amadou, Emmanuelle Gormally

**Affiliations:** 1 Institute for Advanced Biosciences, Joint Research Center Inserm 1209 CNRS UMR 5309 University Grenoble-Alpes, Site Santé, Allée des Alpes, 38700 La Tronche, France; 2 Departement of Prevention Cancer Environment, Centre Léon Bérard, 28 rue Laennec, 69373 Lyon cedex, France; 3 Laboratoire de Biologie Générale, UMRS449 Reproduction et Développement Comparé, Ecole Pratique des Hautes Etudes, 10 place des archives, 69002 Lyon, France; 4 Ecole Supérieure de Biologie-Biochimie-Biotechnologies, Université Catholique de Lyon, 10 place des archives, 69002 Lyon, France

**Keywords:** liver cancer, hepatocellular carcinoma, Africa, aflatoxins, hepatitis B, hepatitis C

## Abstract

Liver cancer (mostly hepatocellular carcinoma, HCC) is both common and highly lethal throughout Africa, in particular in western and middle Africa where HCC is the first cause of cancer death in men and the third in women. In these high-incidence areas, HCC develops mostly early (<50 years), with an aggressive clinical course and frequently without prior diagnosis of liver cirrhosis. The dynamics of African populations predict that the burden of liver cancers will be multiplied by three to four in the next decades unless effective prevention and therapy is achieved. This article outlines a path for significantly curbing the mortality of liver cancer in Africa by combining primary prevention, improved early detection and introduction of innovative and appropriate management strategies.

## Background

Liver cancer is the first cause of death by cancer in men (13% of all cancer deaths) and the third in women (6%) across the African continent [1]. Incidence and mortality rates are almost equal, underlining the lack of curative options. Based on clinical case series, the majority of African liver cancers are hepatocellular carcinomas [2], a cancer that develops from the cells of the liver parenchyma (the hepatocytes), often as a sequel of chronic infectious and inflammatory conditions. Other forms of liver cancer are rare, including intra-hepatic cholangiocarcinoma (CC), a cancer of epithelial cells lining the bile ducts (the cholangiocytes). In Africa, CC is relatively frequent in Egypt but represents less than 5% of the cases documented in clinical series in sub-Saharan Africa [3]. The geographic distribution of liver cancer shows a striking ecological correlation with areas of alternating hot-humid seasons, high endemicity for chronic hepatitis B (HB) and high dietary contamination by aflatoxins, a group of carcinogenic mycotoxins [4–8]. Aflatoxins, chronic HB and chronic hepatitis C infections are all classified as Group 1 carcinogens by the International Agency for Research on Cancer (IARC) [9]. Together, these factors account for over 75% of the risk of hepatocellular carcinoma across sub-Saharan Africa. Other risk factors for hepatocellular carcinoma (HCC) are metabolic disorders, such as obesity, diabetes and other metabolic syndromes.

Most HCCs develop in subjects aged 50 or less, with a mean time between symptoms and diagnosis of just a few weeks indicating that diagnosis occurs late in the course of the disease. Given the current expansion of the population of Africa, the death toll by liver cancer will continue to increase steadily over the next decades, with dramatic human, social and economic consequences, unless a global prevention and management strategy is rolled out.

## Reducing the burden of HCC

From a global perspective, there are four main areas for action in order to decrease the burden of HCC in Africa. These include: 1) preventing hepatitis B virus (HBV) and hepatitis C virus (HCV) infection; 2) mitigating exposure to dietary and metabolic risk factors; 3) treating chronic HBV, HCV and liver disease and 4) improving cancer detection, diagnosis and therapy. Among these measures, neonatal HB vaccination is likely to have the biggest long-term impact. In the meantime, changes in lifestyle may cause an increase in other risk factors, such as metabolic syndromes. Therefore, to have a realistic chance of counterbalancing, the predicted increase due to population expansion, several measures must be combined into a structured action plan. In 2008, we proposed an integrated set of 39 such measures [10]. Table 1 provides a critical discussion and update of these measures in the light of developments over the past decade.

To date, only infant HB vaccination has been rolled out as a structured programme as a part of expanded programs of immunisation using multivalent vaccines usually administered beginning at 6–8 weeks after birth. This achievement has been made possible thanks to the joint efforts of WHO, UNICEF, the Gavi Alliance and African States (http://www.gavi.org/). Neonatal vaccination is remarkably efficient in preventing HB carriage in adulthood, even in the absence of a booster. However, population coverage remains far from complete in over one-third of African countries and the overall coverage at continental level is only about 75%. Thus, due to high natality, the death toll from HCC will continue to grow in the next decades unless better coverage and other prevention measures are efficiently implemented. On the other hand, the precise impact and cost-effectiveness of neonatal HB vaccine on reduction of HCC in adulthood remains to be evaluated in the African context.

In several sub-Saharan countries, changes in agricultural practices aimed at active mitigation of aflatoxin contamination in crops have produced significant socio-economic effects in improving access to market. However, it is not clear whether these measures are sufficient to significantly reduce the levels of chronic exposure that cause liver cancer. Recent evidence from south-eastern Asia shows that rising socio-economic status is associated with diversification of diet types and sources, thus causing less reliance on locally produced contaminated crops and passive reduction of exposure to aflatoxin. Conversely, hypercaloric diets and lifestyles are causing a rapid surge in population obesity, metabolic syndrome and diabetes across the African continent, thus increasing the prevalence of risk factors for liver cancer in both HBV carriers and non-carriers. Overall, these positive and negative trends need to be carefully monitored as many low-resource countries are moving up from low- towards middle-income status.

Access to diagnosis, treatment and palliation is dramatically constrained by economic resources in many African countries. Yet, recent developments in biomarkers for early detection and in therapeutic intervention suggest that an unprecedented window of opportunity exists for screening, early detection and early intervention. Observational studies in populations at risk have shown that surveillance is associated with improved overall survival through detection of HCC at early stages, when patients were eligible to receive potentially curative treatments [11, 12]. Thus, surveillance, screening and therapy must be considered as part of the arsenal for combatting liver cancer in Africa.

The current standard for surveillance is liver ultrasonography at 6-month intervals as recommended by The American Association for the Study of Liver Diseases, The European Association for the Study of the Liver, and The Asia Pacific Association for the Study of the Liver [13–16]. These standards need to be rigorously tested, adapted and evaluated in the African context in order to evaluate their appropriateness and cost-effectiveness in the African context, and ultimately to accelerate their adoption. In the meantime, a rapidly growing number of blood-based biomarkers are being proposed as adjunct or replacement for ultrasonography although their use as a surveillance test for HCC remains controversial. Composite scores, such as GALAD (combining gender, age, AlphaFoetoProtein (AFP), Alpha Foetoprotein P-L3 [a glycoform of AFP found to be more specific for HCC) and des-carboxy-prothrombin]) have shown high accuracy for early detection of HCC [17]. This score can be easily calculated based on patient demographics and cheap blood-based tumour markers using a small volume of peripheral blood, thus offering a practical alternative for surveillance in low-resource contexts, where ultrasound equipment and trained radiologists are scarce. Further research should focus on the identification of low-cost biomarkers, selected for their sensitivity and specificity for early detection of progressive liver disease and liver cancer in African patients.

Whereas access to technology-intensive treatments, such as liver transplant or catheter-based loco-regional treatments is very limited in countries with low or middle resources, percutaneous local ablation is a potentially affordable curative treatment for patients with early stage HCC. Microwave ablation is now the standard modality and is rapidly superseding radiofrequency ablation. On the other hand, rapid advances in clinical trials of kinase inhibitors and immune checkpoint inhibitors are paving the way for new types of molecules that are easy to deliver and may spur access to systemic therapy if available at reasonable prices. The multi-kinase inhibitor sorafenib (targeting VEGFR, PDGFR and Raf family kinases) is the current standard first-line systemic treatment, which has shown prolonged survival of patients with advanced stage HCC in phase III trials. Promising recent results have been recently obtained using immune checkpoint inhibitors in advanced stage HCC [18]. In September 2017, the US FDA granted approval for the use of nivolumab as a second-line treatment for advanced HCC in patients who have been previously treated with sorafenib. These treatments are paving the way for next-generation therapies with improved clinical effects. Further research should focus on the identification of specific drugs targeting the molecular subtypes of HCC that develops in a context of chronic HB and exposure to aflatoxin.

## Conclusions

Altogether, these developments set the scene for a radical paradigm shift in the management of liver cancer in Africa, bringing within the realm of possibility a reduction of mortality of 50% or more over the next generation. This will require careful planning for combining risk reduction, primary prevention, screening and early detection, local ablation and biomarker-based targeted systemic therapies. The key to this approach is to re-design all of the tools and approaches outlined above to adapt them to the specific societal and economic context of African populations in need. In particular, there is an urgent need to mobilise resources in health engineering, connected medicine and biomarker sampling in order to develop devices and solutions that meet the need of these populations. To succeed in rolling out such a programme, there is a critical need for improving clinical capacity and resources in affected countries and regions. This requires advocacy at the local, governmental, regional and global levels in order to develop and deploy effective programmes, to improve access and affordability and to embrace transformative new technologies. At the international level, most efforts are focusing on viral hepatitis, such as those led by the World Hepatitis Alliance. The recent launching of the Global Fund for the Elimination of Viral Hepatitis (EndHEP2030) in November 2017 represents a key step and commitment by the global community sector to mobilise resources towards the achievement of this ambitious goal. This is, however, only one of the components of the global liver cancer problem. Other components, such as screening, diagnosis and treatment must be addressed with the same level of commitment to support capacity building and research on solutions specifically adapted to the needs of the African population. Succeeding in significantly curbing the mortality of liver cancer in Africa may deliver a model for controlling highly lethal cancers in low-resource contexts.

## Conflicts of interest

The authors report no conflicts of interest.

## Funding

The authors did not receive any funding for this work.

## Figures and Tables

**Table 1.** Proposed and updated measures for HCC burden reduction.

Key recommendations by Hainaut and Boyle, 2008 [10]		Critical review and update, 2018
**Development of sustainable childhood vaccination strategies**		**Based on current estimates, we are still half-way in implementing this objective**
	Bring current vaccination trials to final assessment on basis of their disease endpoints	Whereas initial infant vaccination trials in Taiwan gave their first results in the mid-90’s, population-based trials in West Africa (The Gambia), initiated in the mid-eighties, have not yet delivered their final results.
	Monitor long-term protection (vaccine effectiveness) of infant vaccination, assess need for booster vaccines, study interactions with other infections.	Infant HB vaccine is effective against carriage. The effectiveness of HBIG in mother and/or infants needs further evaluation.
	Extend economically sustainable coverage in low-resource areas; Inform and educate populations and stakeholders, obtain participation	Changing perceptions of vaccination are a major threat to universal vaccination programmes.
**Reduction of exposure to aflatoxin**		**Recent results support the feasibility and effectiveness but a global action plan still needs to be rolled out**
	Develop and communicate recommendations on behavioural methods to reduce exposure	The lack of awareness on aflatoxin risk in the population and among stakeholders is still a major obstacle
	Understand mechanisms of synergistic effects between HBV and aflatoxin; assess genetic susceptibility and individual immune responses to the mutagenic effects of aflatoxin	New molecular studies have revealed important clues but there is still a lack of studies using next-generation sequencing to better understand the pan-genomic molecular effects of mutagenesis.
	Stimulate research on substitute crops with less contamination by aflatoxin; Work out economically sustainable models to phase out most contaminated food supplies	Remarkable progress is being made in agricultural research and development. Observational studies in China suggest that decrease in exposure to aflatoxin spontaneously take place as a result of economic growth and changes in lifestyles.
**Reducing factors for NAFLD (Nonalcoholic fatty liver disease)/NASH (Nonalcoholic steatohepatitis)**		**Hypercaloric diet, obesity and physical inactivity are emerging as major risk factors in Africa**
**Controlling HCV-related diseases**		**The availability of treatments for HCV provides unprecedented opportunities**
**Development of registration, early detection, and treatment of hepatocellular carcinoma**		**Registration still covers less than 5% of African population and mortality remains unabated**
	Identify pre-diagnostic and diagnostic markers for screening	New emerging markers need to be tested in prospective studies. Algorithms, hand-held devices and connected medicine should be developed, adapted to African context.
	Study natural history of early disease, and its association with other chronic liver diseases in low-resource contexts	Mechanisms of HCC in patients without pre-existing chronic liver disease/cirrhosis are not well understood.
	Develop liver pathology, biobanking and imaging in low-resource contexts	About 50% of the world’s patients are still diagnosed only on clinical signs, which are misleading.
	Develop preclinical models and clinical trials for targeted therapies and immunotherapies	Current advances are targeted at HCC in high-resource context and not adapted to HCC in low-resource contexts. Yet remarkable opportunities exist for local ablation, targeted therapies and immunotherapies.
	Develop awareness of liver disease, support patients and families, access to palliation	Support for patients and access to palliation are dramatically low in most low-resource contexts
